# Perceived charismatic leadership and work engagement among higher education teachers: differential indirect associations through professional efficacy, belonging, and values

**DOI:** 10.3389/fpsyg.2026.1956141

**Published:** 2026-09-10

**Authors:** Yan Cheng, Junzhang Han, Haibo Liu

**Affiliations:** 1 School of General Education, Shandong Huayu University of Technology, Dezhou, China; 2 Hebei Education Examinations Authority, Shijiazhuang, China; 3 Dezhou Information Engineering Secondary Vocational School, Dezhou, China

**Keywords:** higher education teachers, parallel indirect associations, perceived charismatic leadership, professional belonging, professional efficacy, professional values, work engagement

## Abstract

**Introduction:**

Positive leadership is frequently associated with work engagement, but it remains unclear whether teachers’ perceptions of displayed charismatic leadership behaviors are directly associated with engagement or are linked to it through distinct professional resources. This study examined the indirect associations connecting perceived charismatic leadership with teachers’ work engagement through professional efficacy, professional belonging, and professional values.

**Methods:**

A cross-sectional questionnaire survey was conducted with 248 higher education teachers recruited through convenience sampling in Shandong Province, China. The measurement items were adapted from established scales and localized through translation or wording review, expert review, pilot testing, and revision. Confirmatory factor analysis, structural equation modeling, and bootstrap analysis of specific indirect associations were used to evaluate the proposed model.

**Results:**

Perceived charismatic leadership was positively associated with professional efficacy and professional values but was not uniquely associated with work engagement or professional belonging in the structural model. Professional efficacy and professional values were positively associated with work engagement and yielded significant specific indirect associations between perceived charismatic leadership and work engagement. The corresponding indirect association through professional belonging was not significant.

**Discussion:**

The pattern is consistent with the proposition that perceived charismatic leadership is related to teachers’ work engagement primarily through capability- and meaning-related professional resources. Because the data are cross-sectional and based on adapted self-report measures, the estimates should not be interpreted as evidence of temporal or causal mediation. Subject to longitudinal confirmation, higher education leaders may benefit from pairing inspirational communication with concrete support for capability development and credible professional-purpose formation.

## 1 Introduction

Work engagement has become a central indicator of sustainable performance because it reflects an energetic, dedicated, and absorbed orientation toward work (Mazzetti et al., 2023). In higher education, engaged teachers are more able to sustain teaching quality, research productivity, student support, curriculum improvement, and institutional service under conditions of reform and intensified accountability. These demands make engagement consequential not only for individual well-being but also for institutional continuity and educational quality. Recent higher education research further indicates that engagement can connect leadership and public-service motivation with innovation, citizenship behavior, and job performance (Atiku and Van Wyk, 2024; Guan, 2025; Le, 2026).

Leadership is among the most frequently examined social resources associated with engagement because leaders shape access to information, autonomy, feedback, recognition, developmental opportunities, and the psychological climate of daily work. The job demands-resources model proposes a motivational process through which such resources may support employees’ investment of energy in their roles (Bakker and Demerouti, 2007). Diary, longitudinal, and structural-equation research has associated transformational or engaging leadership with personal resources and engagement under demanding conditions (Tims et al., 2011; Breevaart and Bakker, 2018; Rahmadani et al., 2020). Educational studies similarly link leadership perceptions with teacher outcomes through mastery goals, efficacy, school climate, perceived support, and other psychological mechanisms (Luo et al., 2022; Shao et al., 2025; Li Q. et al., 2026; Li Z. L. et al., 2026).

Charismatic leadership refers to a relational influence process in which followers attribute exceptional credibility and appeal to a leader who communicates a value-laden direction, displays conviction, and models commitment (Tucker, 2003). Although charisma is often treated as a core component of transformational leadership, the constructs are not synonymous. Transformational leadership additionally includes behaviors such as individualized consideration and intellectual stimulation, whereas charismatic leadership centers more narrowly on symbolic direction, identification, conviction, and values-based personal influence (Fuller et al., 1996; DeGroot et al., 2000). In the Chinese transformational-leadership framework developed by Li and Shi (2005), the charismatic dimension is operationalized through professional competence, openness to innovation, work commitment, enthusiasm, and continuous self-development. The present study adopts that culturally grounded, behavior-focused operationalization rather than the entire transformational-leadership construct.

This operational decision also requires caution. Leadership-style research has been criticized for overlap among positively valenced styles and for conflating intended leadership, displayed behavior, follower evaluation, and realized outcomes (Fischer and Sitkin, 2023). The questionnaire used here asked teachers to rate behaviors their leader appeared to display; it did not measure leaders’ intentions or objectively observed behavior. The empirical construct is therefore more precisely described as perceived displayed charismatic leadership. The study does not claim that charisma is superior to servant, authentic, spiritual, situational, or other transformational approaches, nor does it test incremental validity against those styles. It focuses on charisma because the practical problem in autonomous professional settings is whether inspirational credibility is associated with teachers’ engagement after capability-, relationship-, and meaning-related resources are considered. Reliance on inspirational appeal without knowing which professional resources retain unique associations may lead institutions to overestimate the value of charisma alone.

The present study examines three professional resources derived from Li and Yan’s (2018) multidimensional teacher professional-identity framework: professional efficacy, professional belonging, and professional values. Professional efficacy, as operationalized in that framework, concerns perceived professional suitability, responsible task enactment, and confidence in handling teaching work. Professional belonging concerns perceived membership and emotional connection to the school and teaching community. Professional values concern the social meaning, personal worth, contribution, and respect teachers associate with their profession. The dimensions are modeled separately because they represent theoretically different routes: a capability-related route, a relational route, and a meaning-related route.

Existing research supports the relevance of all three resources but does not establish that they make equivalent contributions. Efficacy is a relatively proximal capability belief and has been associated with both leadership perceptions and teacher engagement (Xie and Wang, 2025; Zhang et al., 2026; Gao et al., 2026). Belonging is socially embedded and depends on collegial relationships, recognition, voice, institutional support, and leadership rather than on one leader’s behavior alone (Wator et al., 2025; Allen et al., 2025; Bjorklund and Karnopp, 2026). Values offer a meaning-related route: longitudinal evidence has linked leadership perceptions with later engagement through value congruence, while mission valence has been examined as an intervening motivational condition (Mastrorilli et al., 2026; Xie et al., 2026). Because these resources are correlated, testing them in separate models could obscure whether each retains a unique association with engagement. Their simultaneous inclusion provides a more stringent comparison, although the present cross-sectional design cannot establish temporal mediation.

Accordingly, this study asks whether perceived charismatic leadership has a unique direct association with higher education teachers’ work engagement, whether it is associated with each professional resource, and whether the data support distinct specific indirect associations through efficacy, belonging, and values. The study contributes by delimiting the measured leadership construct, disaggregating professional identity into three resources, and testing their unique statistical roles in Chinese higher education. Its contribution is explanatory differentiation rather than a claim that charismatic leadership is the strongest or causally superior leadership style.

## 2 Literature review and hypotheses development

### 2.1 Integrated theoretical framework: leadership and resource conversion

The job demands-resources (JD-R) model provides the principal resource-based framework for the proposed associations. Job resources support goal attainment, reduce the psychological costs of job demands, and stimulate learning and development. Leadership may be experienced as a social job resource because leaders are associated with autonomy, clarity, feedback, recognition, and access to developmental support (Bakker and Demerouti, 2007). Meta-analytic evidence identifies resources as important correlates of work engagement, and recent reviews likewise emphasize configurations of demands and resources in teacher well-being (Mazzetti et al., 2023; Moens et al., 2026). JD-R was selected because it explicitly connects contextual resources with personal resources and work engagement, matching the study’s comparison of three professional-resource dimensions.

JD-R is not the only account of engagement. Kahn (1990) proposed that personal engagement depends on psychological meaningfulness, safety, and availability, and May et al. (2004) provided empirical support for these psychological conditions. This perspective is complementary to the present framework: professional values are conceptually adjacent to meaningfulness, professional belonging may contribute to safety, and professional efficacy may relate to perceived availability. These constructs are not interchangeable, however, and the study did not directly measure Kahn’s three states. The use of JD-R therefore reflects the resource-conversion research question rather than an assumption that psychological-state explanations are unimportant or that the three professional-identity dimensions substitute for meaningfulness, safety, and availability.

Within the proposed framework, social cognitive theory explains why a perceived leadership resource may be associated with professional efficacy. Efficacy beliefs develop through mastery experience, vicarious learning, verbal persuasion, and interpretations of challenge (Bandura, 1997). A leader perceived as competent and enthusiastic may provide models and confidence-relevant cues, while developmental feedback and manageable framing may support capability beliefs. This reasoning positions professional efficacy as the capability-related resource in the hypothesized ordering.

Capability does not exhaust the possible motivational relevance of leadership. Value-based and fit perspectives suggest that people invest more deeply when work expresses important personal and collective values. Leadership communication may connect everyday tasks with wider educational missions and perceived value congruence. Longitudinal evidence indicates that value congruence can link leadership perceptions with subsequent work engagement (Mastrorilli et al., 2026), while mission-valence research identifies valued social purpose as a possible intervening condition between servant leadership and engagement (Xie et al., 2026). These findings support professional values as the meaning-related resource in the model.

Professional belonging represents the relational resource. Belonging may provide social safety, identity continuity, and a sense that one’s contribution is recognized. Teacher-belonging research shows, however, that this resource is jointly associated with collegial interaction, collective values, recognition, institutional stability, voice, and supportive leadership (Wator et al., 2025; Allen et al., 2025). Perceived charismatic behavior may be related to this environment, but belonging is likely to depend more heavily on distributed social and institutional conditions than are capability or meaning. The model therefore allows the three associations to differ rather than assuming that all positive professional perceptions operate identically.

### 2.2 Charismatic leadership and work engagement

In the present study, perceived charismatic leadership denotes teachers’ ratings of displayed professional competence, openness to innovation, enthusiasm, commitment, and self-development. This is narrower than a complete transformational-leadership profile and somewhat more behavior-specific than classical accounts centered on extraordinary personal attribution. The distinction is important because meta-analytic reviews show that associations involving charisma vary with the outcome, level of analysis, source of measurement, and study design (Fuller et al., 1996; DeGroot et al., 2000). In universities, professional credibility may nevertheless be especially salient because teachers possess substantial expertise and autonomy.

A broad leadership literature reports positive associations between leadership perceptions and engagement. Transformational leadership has been linked with followers’ daily engagement partly through optimism and other resources, while engaging leadership has been associated with need satisfaction and job resources (Tims et al., 2011; Rahmadani et al., 2020). Higher education studies similarly connect leadership practices with engagement through job conditions and psychological resources (Atiku and Van Wyk, 2024). Charismatic communication has also been associated with organizational engagement through psychological-need satisfaction (Liang et al., 2026). These findings motivate a positive hypothesis but do not demonstrate that charisma is empirically distinct from every other positive leadership style or that it necessarily precedes engagement.

Direct leadership coefficients may become weak once more proximal resources are included. Studies in universities and other organizations identify autonomy, psychological empowerment, and related resources as statistical intervening variables (Gözükara and Simsek, 2015; Lai et al., 2020; Abdulrab et al., 2025). The present study therefore tests the theoretically expected positive association while recognizing that its unique coefficient may depend on the professional resources included in the same model.

H1: Perceived charismatic leadership is positively associated with higher education teachers’ work engagement.

### 2.3 Charismatic leadership and professional efficacy

Professional efficacy refers to teachers’ perceived capability to perform teaching work seriously, confidently, and effectively. It is derived here from the professional-identity framework of Li and Yan (2018), whereas much of the international literature uses the related term teacher self-efficacy. Both constructs concern perceived capability, but the present measure is anchored specifically in teachers’ professional suitability, task completion, and confidence in handling teaching work.

Perceived charismatic leadership is theorized to be associated with professional efficacy because displayed competence, encouragement, cognitive framing, and access to developmental opportunities may provide confidence-relevant cues. Leaders who demonstrate professional competence and enthusiasm may offer vicarious evidence that demanding work is manageable, while confidence-oriented communication may reduce uncertainty. Research across educational leadership forms has reported teacher efficacy as an intervening variable linking leadership perceptions with work attitudes and performance (Luo et al., 2022; Zhang et al., 2025; Limon et al., 2026).

Recent studies provide relevant evidence while using designs and constructs distinct from the present study. Self-efficacy statistically mediated the association between paternalistic leadership and work engagement among medical university teachers (Xie and Wang, 2025). Other studies reported indirect relationships involving authentic leadership, school climate, teacher efficacy, calling, transformational leadership, inclusive leadership, and thriving at work (Shao et al., 2025; Zhang et al., 2026; Gao et al., 2026). These findings support professional efficacy as a plausible correlate in the hypothesized ordering; they do not establish the ordering in the present cross-sectional data.

H2a: Perceived charismatic leadership is positively associated with professional efficacy.

### 2.4 Charismatic leadership and professional belonging

Professional belonging refers to teachers’ perceived membership in the school and teaching community and the extent to which personal development is connected with collective development. Belonging can strengthen social identity, reduce isolation, and encourage participation beyond narrowly defined tasks. Teacher-belonging research therefore treats it as an important condition for well-being, retention, and professional participation (Wator et al., 2025).

Perceived charismatic leadership is theorized to be positively associated with belonging because shared direction, respectful communication, collective identification, and acknowledgment of individual contributions may coincide with a stronger sense of membership. Trust in leaders has been associated with work engagement and organizational outcomes (Olvera et al., 2024). In educational settings, opportunities for voice and having one’s opinions acknowledged may also be important correlates of teacher belonging (Bjorklund and Karnopp, 2026). Research during school reform further suggests that charismatic-leadership perceptions and teacher engagement can be associated with organizational loyalty (Özdemir et al., 2024).

At the same time, belonging is a socially distributed experience. A large qualitative survey of Australian teachers identified interpersonal relationships, collaboration, professional growth, institutional factors, recognition, and external networks as major belonging conditions (Allen et al., 2025). A scoping review similarly identified collegial interaction, collective values, leadership, recognition, and teacher-student relationships as central themes (Wator et al., 2025). Evidence concerning voice and counted opinions further indicates that participatory structures are relevant alongside focal-leader perceptions (Bjorklund and Karnopp, 2026). On balance, a positive association between perceived charismatic leadership and professional belonging is theoretically expected.

H2b: Perceived charismatic leadership is positively associated with professional belonging.

### 2.5 Charismatic leadership and professional values

Professional values refer to teachers’ beliefs that their work contributes to social development, supports students’ growth, realizes personal life value, and earns social respect. They therefore capture not only preferences for job characteristics but also the moral and social meaning attributed to teaching. Contemporary work-values research emphasizes that values shape occupational choice, fit, and sustained commitment (Heimpel et al., 2026).

Perceived charismatic leadership is theorized to be associated with professional values because communication about why teaching matters, visible commitment to educational purposes, and links between everyday responsibilities and wider goals may coincide with stronger meaning attribution. Purposeful communication may be related to whether demanding tasks are interpreted as contributions rather than mere obligations. Evidence concerning value congruence and later engagement supports the plausibility of this meaning-related account (Mastrorilli et al., 2026).

Research outside education also shows associations between professional values and professional quality of life and has examined mission valence as an intervening condition in leadership-engagement relationships (Ni et al., 2025; Xie et al., 2026). In Chinese higher education, public-service motivation is relevant because educational work is closely tied to social contribution and student development, and engagement has been associated with the link between such motivation and performance (Guan, 2025). A positive association between perceived charismatic leadership and teachers’ professional values is therefore expected.

H2c: Perceived charismatic leadership is positively associated with professional values.

### 2.6 Professional resources and work engagement

#### 2.6.1 Professional efficacy and work engagement

Professional efficacy is expected to be positively associated with engagement because employees may invest more energy when they believe that effort can produce successful performance. Teachers with stronger efficacy may be more likely to approach difficult tasks as challenges, persist after setbacks, and maintain concentration. This capability belief is therefore theoretically more proximal to work behavior than a general positive attitude (Bandura, 1997).

Empirical research repeatedly reports associations between efficacy and engagement. Prior studies have modeled self-efficacy as an intervening variable in leadership-engagement relationships among medical university teachers and broader teacher samples (Tripiana and Llorens, 2015; Xie and Wang, 2025; Zhang et al., 2026). Evidence from early childhood education also describes teacher self-efficacy and work engagement as interconnected resources in leadership processes (Marçalo et al., 2026).

H3a: Professional efficacy is positively associated with work engagement.

#### 2.6.2 Professional belonging and work engagement

Professional belonging is expected to be positively associated with engagement because people may be more willing to contribute when they feel included, recognized, and connected with a valued community. Relational security may make effort socially meaningful and may accompany continued participation during periods of change. Reviews of teacher belonging emphasize its relevance to identity, well-being, and participation in school life (Wator et al., 2025).

However, belonging may not automatically become task-focused energy. Its relationship with engagement may depend on whether membership is accompanied by voice, fairness, trust, collaboration, and opportunities to contribute. Allen et al. (2025) showed that belonging arises from multiple interpersonal and institutional conditions, while Bjorklund and Karnopp (2026) highlighted the importance of having one’s opinion counted. Despite these contingencies, a positive relationship is theoretically expected.

H3b: Professional belonging is positively associated with work engagement.

#### 2.6.3 Professional values and work engagement

Professional values are expected to be positively associated with engagement because meaning and value alignment can make work activities personally important. When teachers believe that their work advances students and society, they may have a stronger reason to sustain effort despite pressure. Professional values may therefore supply motivational direction even when external rewards are limited.

Evidence from value-congruence and mission-valence research supports the plausibility of this association. A two-wave study found that leadership-related value congruence predicted subsequent work engagement (Mastrorilli et al., 2026), while mission valence and motivation were modeled as intervening variables between servant leadership and volunteer work engagement (Xie et al., 2026). Professional values have also been associated with professional quality of life in demanding service occupations (Ni et al., 2025).

H3c: Professional values are positively associated with work engagement.

### 2.7 Parallel indirect associations through professional resources

A parallel indirect-association model is appropriate because perceived charismatic leadership may be related to several professional resources at the same time, while those resources may differ in their unique associations with engagement. Research increasingly shows that leadership-outcome relationships are statistically linked with multiple and non-equivalent intervening variables, including job conditions, efficacy beliefs, psychological empowerment, perceived support, and value congruence (Atiku and Van Wyk, 2024; Abdulrab et al., 2025; Mastrorilli et al., 2026).

Professional efficacy is expected to be comparatively proximal because it concerns whether teachers believe that they can perform demanding tasks. Professional values provide a meaning-related route by clarifying why the work deserves investment. Professional belonging provides a relational route but may depend more heavily on organizational climate and collegial conditions before it is associated with task-focused engagement. Estimating the three variables together tests whether each proposed route explains unique covariance rather than merely reflecting a common positive professional orientation.

**FIGURE 1:**
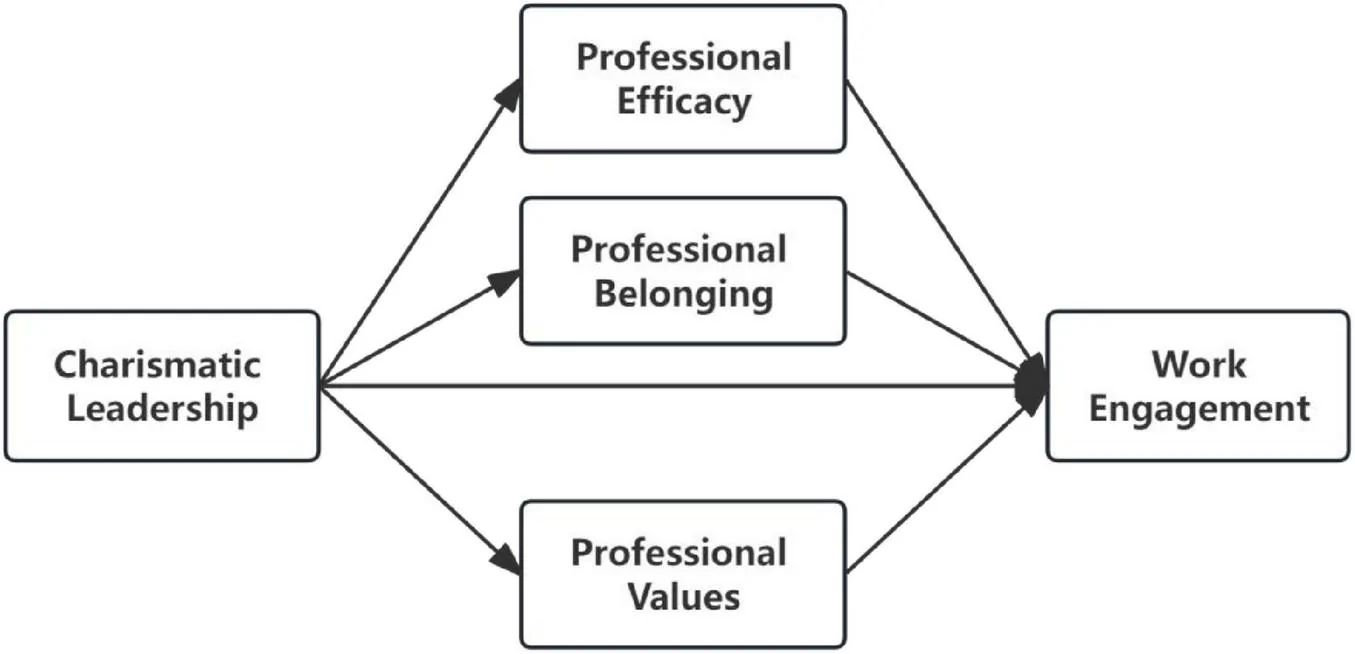
Conceptual model and hypothesized associations. Perceived CHL = perceived charismatic leadership. Professional efficacy, professional belonging, and professional values were tested as proposed parallel mediators. Arrows indicate the hypothesized theoretical ordering; they do not represent causal effects established by the cross-sectional design.

The conceptual model is presented in Figure 1. It specifies one direct association and three simultaneous specific indirect associations through professional efficacy, professional belonging, and professional values. The arrows represent the hypothesized theoretical ordering; the cross-sectional data evaluate whether the covariance pattern is consistent with that ordering, not whether temporal or causal mediation occurred.

H4a: Perceived charismatic leadership has a positive specific indirect association with work engagement through professional efficacy.

H4b: Perceived charismatic leadership has a positive specific indirect association with work engagement through professional belonging.

H4c: Perceived charismatic leadership has a positive specific indirect association with work engagement through professional values.

## 3 Methodology

### 3.1 Research design

This study adopted a quantitative, cross-sectional correlational survey design to examine associations among perceived charismatic leadership, professional efficacy, professional belonging, professional values, and work engagement among higher education teachers. The design permitted estimation of relationships among latent constructs and comparison of theoretically distinct specific indirect associations within one model. It did not permit verification of temporal precedence or causal mediation.

The unit of analysis was the individual higher education teacher. Perceived charismatic leadership was specified as the focal predictor, work engagement as the outcome, and professional efficacy, professional belonging, and professional values as three proposed parallel mediators. Although the three mediators were adapted from a multidimensional teacher professional-identity framework, they were modeled as separate first-order constructs rather than as indicators of one higher-order professional-identity variable. This specification compared capability-related, relational, and meaning-related resources and estimated whether each accounted for unique covariance with work engagement.

Covariance-based structural equation modeling was used to evaluate the hypothesized model. The analytical procedure incorporated assessment of the measurement model, common method variance, construct reliability and validity, structural model fit, direct associations, and specific indirect estimates. The conceptual model contained five latent constructs measured by 18 observed indicators.

### 3.2 Participants and sampling

The target population consisted of full-time and part-time teachers with teaching experience in higher education institutions in China. To be eligible for participation, respondents were required to have current or recent higher education teaching experience, be able to evaluate leadership behaviors within their work context, and be willing to report their professional perceptions and work engagement. Participants who did not meet these inclusion criteria were excluded from the final analysis.

Convenience sampling was used because a complete probability sampling frame of higher education teachers was unavailable. Recruitment links were distributed through professional contacts across five higher education institutions and disciplinary networks in Shandong Province. No institutional quota or probability-selection procedure was applied.

Participants were recruited from two public undergraduate institutions, two private undergraduate institutions, and one higher vocational institution. To protect institutional confidentiality, the institutions are identified as Institutions A–E. Among the 248 respondents retained for analysis, 56 (22.6%) were from Institution A, 51 (20.6%) from Institution B, 49 (19.8%) from Institution C, 47 (19.0%) from Institution D, and 45 (18.1%) from Institution E. Institution-specific sample sizes therefore ranged from 45 to 56. In terms of institutional type, 107 respondents (43.1%) were employed at public undergraduate institutions, 96 (38.7%) at private undergraduate institutions, and 45 (18.1%) at a higher vocational institution. Because respondents were nested within a limited number of institutions, possible shared institutional influences should be considered when interpreting the individual-level results.

Data were collected between September 2025 and February 2026 through Wenjuanxing, an online survey platform in China. Before accessing the formal questionnaire, potential participants were presented with an electronic information sheet explaining the purpose of the study, the voluntary nature of participation, the confidentiality of their responses, the academic use of the collected data, and their right to discontinue the survey at any time before submission without penalty. Participants were also informed that the questionnaire would not collect directly identifying personal information.

Only respondents who indicated that they had read the research information and voluntarily agreed to participate were permitted to proceed to the formal questionnaire. Electronic informed consent was therefore obtained from all participants included in the study. Participation was entirely voluntary, and respondents could exit the questionnaire before submission without providing a reason.

A total of 310 questionnaires were initially received. After applying the data-screening criteria described in Section 3.3, 62 responses were excluded because they did not satisfy the eligibility or data-quality requirements. The final analytical sample therefore comprised 248 valid responses, representing a retention rate of 80.0%. The same 248 cases were used consistently in the descriptive analysis, confirmatory factor analysis, common method assessment, reliability and validity tests, correlation analysis, structural equation modeling, and bootstrap analysis of specific indirect associations.

As shown in Table 1, the final sample consisted of 64 male teachers (25.8%) and 184 female teachers (74.2%). Regarding age, 121 participants (48.8%) were aged 30 years or below, 117 participants (47.2%) were aged between 31 and 50 years, and 10 participants (4.0%) were above 50 years old. In terms of professional title, 207 participants (83.5%) were lecturers or below, whereas 41 participants (16.5%) were associate professors or above. The participants represented several disciplinary areas, including engineering (33.1%), economics and management (21.0%), arts and other fields (19.0%), foreign languages (15.3%), and humanities (11.7%). Table 1 also reports the institutional distribution and institutional types represented in the final sample.

**TABLE 1:** Participant characteristics.

Variable	Category	n	Percentage (%)
Gender	Male	64	25.8
	Female	184	74.2
Age	30 years old and below	121	48.8
	31–50 years old	117	47.2
	Above 50 years old	10	4.0
Professional title	Lecturer and below	207	83.5
	Associate professor and above	41	16.5
Discipline	Arts and other fields	47	19.0
	Foreign languages	38	15.3
	Economics and management	52	21.0
	Humanities	29	11.7
	Engineering	82	33.1
Institution	Institution A	56	22.6
	Institution B	51	20.6
	Institution C	49	19.8
	Institution D	47	19.0
	Institution E	45	18.1
Institution type	Public undergraduate institution	107	43.1
	Private undergraduate institution	96	38.7
	Higher vocational institution	45	18.1

N = 248. Institution names are anonymized as Institutions A–E. Percentages may not sum to exactly 100% because of rounding.

### 3.3 Data screening and final sample

Before the formal analyses, all submitted questionnaires were screened for participant eligibility, response quality, and response consistency. All questionnaire items were mandatory on Wenjuanxing; consequently, no item-level missing values were present. Responses were excluded when they displayed clear repetitive or patterned responding, were completed in less than 50 s, or did not satisfy the inclusion criteria for higher education teachers. The 50-s threshold was used as a conservative lower bound because it allowed less than 2.8 s per substantive item before demographic questions, making attentive reading of the 18 items implausible.

A total of 310 questionnaires were submitted. The screening criteria were applied sequentially so that each excluded questionnaire was assigned only one primary exclusion reason. Fourteen questionnaires were excluded because the respondents did not meet the eligibility criteria, 27 were excluded because the completion time was less than 50 s, and 21 were excluded because they displayed clearly repetitive or patterned responses. Consequently, 62 questionnaires were excluded and 248 valid questionnaires were retained, representing a retention rate of 80.0%. No additional cases were selectively removed during the measurement- or structural-model analyses, and the same 248 cases were used throughout.

For all 310 submitted questionnaires, the mean completion time was 274.8 s (SD = 146.2), and the median was 238 s (IQR = 172–356; range = 31–1,425). Among the 248 questionnaires retained for analysis, the mean completion time was 286.4 s (SD = 131.7), and the median was 254 s (IQR = 188–347; range = 79–1,120). The 27 questionnaires excluded under the completion-time criterion had a mean completion time of 41.2 s (SD = 6.3) and a median of 42 s (IQR = 37–46; range = 31–49).

The final sample provided 13.8 cases per observed indicator for the five-factor, 18-indicator model. In addition, an RMSEA-based model-fit power calculation following MacCallum et al. (1996), with N = 248, df = 128, alpha = 0.05, a close-fit null of RMSEA = 0.05, and an alternative of RMSEA = 0.08, yielded estimated power of 0.997. This is a model-level assessment rather than proof of power for every indirect association; the precision of the specific indirect estimates was evaluated separately with 5,000 bootstrap resamples. Sample adequacy was also judged from the absence of inadmissible solutions, the factor loadings, standard errors, fit indices, and bootstrap confidence intervals rather than from a single rule of thumb.

### 3.4 Instruments

All constructs were measured using items adapted from established scales and localized to the context of Chinese higher education. The localization followed a multi-stage procedure. For items originating from English-language measures, the original items were translated into Chinese and subsequently back-translated into English. The original and back-translated versions were compared to identify and resolve discrepancies in meaning. For items derived from Chinese-language source scales, the wording was reviewed and adjusted to reflect the occupational language and leadership context of higher education teachers.

Experts were invited to review the clarity, construct relevance, semantic equivalence, and contextual appropriateness of the adapted items. A pilot test was subsequently conducted before the formal survey. Feedback obtained from the expert review and pilot test was used to revise ambiguous, repetitive, or contextually inappropriate expressions. These procedures were intended to preserve the conceptual meaning of the source measures while improving the clarity, comprehensibility, and contextual relevance of the questionnaire.

All items were measured on a five-point Likert scale ranging from 1 = strongly disagree to 5 = strongly agree. Higher scores indicated higher levels of the corresponding construct. As shown in Table 2, the final instrument contained 18 items measuring five constructs.

**TABLE 2:** Localized measurement items.

Construct	Item code	Measurement item
Perceived Charismatic Leadership	CHL1	The leader demonstrates strong professional competence.
	CHL2	The leader is open-minded and has strong innovative thinking.
	CHL3	The leader is highly committed to work and maintains a strong sense of enthusiasm.
	CHL4	The leader continuously engages in self-learning to improve and develop themselves.
Professional Values	PV1	My work plays an important role in promoting social development.
	PV2	My work is important for promoting students’ growth and development.
	PV3	Engaging in the teaching profession enables me to realize my life value.
	PV4	Working as a teacher allows me to gain respect from others.
Professional Belonging	PB1	I consider myself a member of the teaching community, and my development is closely connected with the development of this community.
	PB2	I often feel that I am a member of my school, and my development is closely connected with the development of the school.
	PB3	I care about others’ evaluations of my school, and when I see or hear positive or negative comments about it, I feel a shared sense of honor or shame.
Professional Efficacy	PE1	I believe that my personality is suitable for being a teacher.
	PE2	I am able to complete my current teaching work seriously and in a timely manner.
	PE3	I usually feel confident when dealing with teaching work.
Work Engagement	WE1	I can work continuously for a long period of time.
	WE2	Time always passes quickly when I am working.
	WE3	When I am working, I forget everything around me.
	WE4	I am rarely distracted while working.

All items were rated from 1 = strongly disagree to 5 = strongly agree. The English wording is presented for manuscript reporting; the administered questionnaire used localized Chinese expressions. The four work-engagement items do not constitute the complete UWES-9 and emphasize sustained involvement and absorption.

#### 3.4.1 Perceived charismatic leadership

Perceived charismatic leadership was measured with four items adapted from the charismatic leadership dimension of Li and Shi’s (2005) Chinese transformational-leadership scale. Their broader framework includes charismatic leadership alongside other dimensions such as moral modeling and individualized consideration; the present measure should therefore not be interpreted as a measure of the complete transformational-leadership construct.

Only the charismatic dimension was retained because the research question concerned displayed professional competence, openness to innovation, work commitment, enthusiasm, and continuous self-development. Item wording was localized for higher education. Because teachers reported what their leaders appeared to demonstrate, the scores represent follower perceptions of displayed behavior, not leader intentions, objectively coded actions, or realized causal effects. Sample items included ‘The leader demonstrates strong professional competence’ and ‘The leader is open-minded and has strong innovative thinking.’

#### 3.4.2 Professional efficacy

Professional efficacy was measured with three items drawn together from the professional-efficacy component of Li and Yan’s (2018) multidimensional teacher professional-identity scale. In that validated source framework, the component is broader than task-specific self-efficacy: it combines perceived suitability for the profession, responsible and timely task enactment, and confidence in handling teaching work. The three retained items were not assembled from unrelated scales.

Accordingly, professional efficacy in this study is an identity-embedded, efficacy-related professional perception rather than a direct substitute for Bandura-style, task-specific teacher self-efficacy. PE3 most directly expresses confidence, whereas PE1 and PE2 also contain person-profession fit and conscientious enactment content. The distinct CFA factor, alpha, composite reliability, and AVE support internal coherence in this sample, but they do not eliminate this content-validity limitation. A sample item was ‘I usually feel confident when dealing with teaching work.’

#### 3.4.3 Professional belonging

Professional belonging was measured using three items adapted from Li and Yan’s (2018) teacher professional identity framework. The items assessed teachers’ perceived membership in the teaching community and their institution, as well as the perceived connection between their personal development and the development of the wider professional or institutional community.

The construct therefore represented a relational professional resource rather than a general measure of organizational commitment. A sample item was “I often feel that I am a member of my school, and my development is closely connected with the development of the school.”

#### 3.4.4 Professional values

Professional values were measured with four items adapted from Li and Yan (2018). The items reflected teachers’ beliefs that their work contributes to social development, promotes students’ growth, enables the realization of personal life value, and generates occupational respect.

Professional values were conceptualized as a meaning-based resource because they captured the social and personal significance teachers attributed to their profession. A sample item was “My work is important for promoting students’ growth and development.”

#### 3.4.5 Work engagement

Work engagement was assessed with four localized items selected with reference to Schaufeli et al.’s (2006) UWES framework. One item reflects sustained involvement or persistence, while the remaining items primarily reflect absorption, subjective time passage, and resistance to distraction.

The measure was not treated as the validated UWES-9 and does not provide balanced coverage of vigor, dedication, and absorption. In particular, dedication is not directly represented and vigor is represented only indirectly by sustained work. The four items were therefore modeled as a single adapted work-engagement indicator set, and no dimension-specific claims were made. This choice offers a brief assessment of sustained and absorbed work involvement but narrows the content domain of the outcome.

Sample items included ‘I can work continuously for a long period of time’ and ‘When I am working, I forget everything around me.’ The measurement limitation is considered when interpreting the findings and is stated explicitly in the limitations section.

### 3.5 Data analysis

Data were analyzed using SPSS 26.0 and AMOS 26.0. Because all questionnaire items were mandatory, no item-level missing-data treatment was required. Before model estimation, the univariate distributions of the 18 observed indicators were examined using skewness and kurtosis. Skewness values ranged from −1.024 to 0.358, while kurtosis values ranged from −0.714 to 0.732. These results indicated no serious departures from univariate normality. Parameters in the confirmatory factor analysis and structural equation models were estimated using maximum likelihood estimation. The analysis then proceeded through five stages.

First, descriptive statistics were used to summarize the demographic characteristics of the final sample. Frequencies and percentages were calculated for gender, age, professional title, and disciplinary background. All descriptive results were based on the same final analytical sample of 248 participants.

Second, confirmatory factor analysis was conducted to assess the measurement structure. The hypothesized five-factor model was compared with models that merged each pair of professional-resource constructs, merged professional efficacy with work engagement, combined all professional-resource items, combined all non-leadership items, and loaded all indicators on one factor. Model fit was evaluated using chi-square, chi-square divided by degrees of freedom, GFI, CFI, TLI, RMSEA with its 90% confidence interval, SRMR, AIC, and Chi-square difference tests. The indices were interpreted collectively rather than according to one isolated cutoff (Hu and Bentler, 1999).

Third, common method variance was assessed using two complementary statistical procedures. The hypothesized five-factor measurement model was first compared with a one-factor model. An unmeasured latent method construct model was then estimated and compared with the baseline five-factor model. These procedures were used because common method variance should not be evaluated solely through Harman’s single-factor test (Podsakoff et al., 2003; Richardson et al., 2009).

Fourth, the reliability and validity of the measurement model were evaluated. Internal consistency was assessed using Cronbach’s alpha and composite reliability, and convergent validity was examined through standardized factor loadings and average variance extracted. Discriminant validity was evaluated with both the Fornell-Larcker criterion and heterotrait-monotrait ratios (HTMT), using 0.85 as a conservative reference value (Fornell and Larcker, 1981; Henseler et al., 2015).

Fifth, structural equation modeling was used to estimate the hypothesized direct associations. The three specific indirect associations between perceived charismatic leadership and work engagement were evaluated with 5,000 bootstrap resamples, and both bias-corrected and percentile 95% confidence intervals were reported. An indirect estimate was considered statistically distinguishable from zero when its interval excluded zero (Hayes, 2022). Because the variables were measured at one time point, these estimates are reported as cross-sectional indirect associations and do not establish temporal mediation.

All descriptive, measurement-model, structural-model, correlation, reliability, validity, and indirect-association analyses were based on the same final sample of 248 participants.

## 4 Results

### 4.1 Measurement-model comparisons and common method assessment

The hypothesized five-factor structure was first compared with seven theoretically plausible collapsed-factor models. Because all focal variables were obtained from the same respondents, common method covariance was then examined by comparing the five-factor baseline with both a one-factor model and a model containing an unmeasured latent method construct. All analyses used the same 248 cases.

As shown in Table 3, the five-factor model fit the data well: chi-square = 178.526, df = 125, Chi-square/df = 1.428, CFI = 0.979, TLI = 0.975, RMSEA = 0.042, 90% CI [0.027, 0.055], and SRMR = 0.0369. Every collapsed-factor alternative fit significantly worse (all Chi-square-difference *p* values < 0.001). Even the closest alternative, which combined professional efficacy with work engagement, had lower fit (Chi-square = 253.83, df = 129, CFI = 0.952, TLI = 0.943, RMSEA = 0.063, SRMR = 0.046; delta chi-square = 75.30, delta df = 4). Models that combined the professional-resource dimensions performed substantially worse. These comparisons support retaining five factors while recognizing the conceptual relatedness of the professional resources.

**TABLE 3:** Comparison of competing measurement models (CFA).

ID	Model specification	χ ^2^	*df*	χ ^2^/*df*	CFI	TLI	RMSEA [90% CI]	SRMR	AIC	Δχ ^2^	Δ *df*
M1	Five-factor model (Hypothesized)	178.53	125	1.43	0.979	0.975	0.042 [0.027, 0.055]	0.037	270.53	–	–
M2	Four-factor model (PE + PB combined)	759.85	129	5.89	0.758	0.713	0.141 [0.131, 0.151]	0.219	843.85	581.32	4
M3	Four-factor model (PE + PV combined)	306.20	129	2.37	0.932	0.919	0.075 [0.064, 0.085]	0.049	390.20	127.68	4
M4	Four-factor model (PB + PV combined)	856.44	129	6.64	0.721	0.669	0.151 [0.142, 0.161]	0.231	940.44	677.91	4
M5	Three-factor model (PE + PB + PV combined)	1383.18	132	10.48	0.521	0.444	0.196 [0.187, 0.205]	0.284	1461.18	1204.65	7
M6	Four-factor model (PE + WE combined)	253.83	129	1.97	0.952	0.943	0.063 [0.051, 0.074]	0.046	337.83	75.30	4
M7	One-factor model (All combined)	1281.47	135	9.49	0.561	0.502	0.185 [0.176, 0.195]	0.156	1353.47	1102.94	10
M8	Two-factor model (CHL vs. Others combined)	887.10	134	6.62	0.711	0.67	0.151 [0.142, 0.160]	0.116	961.10	708.58	9

*N* = 248. CHL, perceived charismatic leadership; PV, professional values; PB, professional belonging; PE, professional efficacy; WE, work engagement. The hypothesized five-factor model (M1) served as the baseline for comparison. Δχ^2^ and Δdf represent the differences in Chi-square and degrees of freedom, respectively, against the baseline model. All Chi-square difference tests were significant at *p* < 0.001, indicating a significantly worse fit for all competing models compared to the hypothesized five-factor model.

The unmeasured latent method construct (ULMC) model was then estimated to assess covariance potentially attributable to the common response method. As shown in Table 4, it yielded Chi-square = 123.919, df = 107, Chi-square/df = 1.158, GFI = 0.945, CFI = 0.994, TLI = 0.991, RMSEA = 0.025, and SRMR = 0.0273. Relative to the already satisfactory five-factor baseline, GFI increased by 0.019, CFI by 0.015, and TLI by 0.016, while RMSEA and SRMR decreased by 0.017 and approximately 0.010, respectively.

**TABLE 4:** ULMC test results.

Model	χ ^2^	df	χ ^2^/df	GFI	CFI	TLI	RMSEA	SRMR	△GFI	△CFI	△TLI	△RMSEA	△SRMR
M1: Five-factor baseline	178.526	125	1.428	0.926	0.979	0.975	0.042	0.0369	–	–	–	–	–
M2: ULMC model	123.919	107	1.158	0.945	0.994	0.991	0.025	0.0273	0.019	0.015	0.016	−0.017	−0.010

M1 = baseline five-factor measurement model; M2 = model including an unmeasured latent method construct. Change values are relative to M1.

The poor one-factor model indicates that one general factor did not dominate the item covariance. However, the improvement after adding a latent method factor indicates that some method-related covariance cannot be ruled out. The ULMC analysis is therefore treated as a sensitivity check rather than proof that common method bias is absent, and the single-source associations are interpreted cautiously.

### 4.2 Reliability and convergent validity

As shown in Table 5, standardized factor loadings ranged from 0.675 to 0.910 and were statistically significant. Cronbach’s alpha values ranged from 0.836 to 0.896, and composite reliability values ranged from 0.848 to 0.897. These values exceeded commonly accepted thresholds for internal consistency.

**TABLE 5:** Reliability and convergent validity of the measurement model.

Constructs	Indicators	*Mean*	*SD*	*B*	*SE*	*z*	*p*	β	α	CR	AVE
Perceived CHL	CHL1	2.68	0.775	1				0.678	0.846	0.848	0.584
	CHL2	2.69	0.772	1.203	0.113	10.653	***	0.818			
	CHL3	2.59	0.795	1.194	0.115	10.408	***	0.789			
	CHL4	2.58	0.721	1.051	0.103	10.179	***	0.765			
Professional values	PV1	3.79	1.02	1				0.784	0.878	0.880	0.647
	PV2	3.92	0.988	1.02	0.074	13.796	***	0.825			
	PV3	3.83	1.049	1.129	0.078	14.465	***	0.861			
	PV4	3.72	1.068	0.992	0.082	12.164	***	0.743			
Professional belonging	PB1	2.43	0.802	1				0.91	0.896	0.897	0.744
	PB2	2.42	0.775	0.924	0.053	17.428	***	0.87			
	PB3	2.39	0.766	0.845	0.053	15.813	***	0.805			
Professional efficacy	PE1	3.77	1.112	1				0.675	0.836	0.850	0.656
	PE2	4.11	0.948	1.078	0.092	11.736	***	0.853			
	PE3	3.86	1.013	1.195	0.099	12.051	***	0.886			
Work engagement	WE1	3.56	1.196	1				0.728	0.873	0.878	0.643
	WE2	3.84	1.06	1.014	0.08	12.673	***	0.833			
	WE3	3.63	1.025	0.998	0.077	12.884	***	0.847			
	WE4	3.58	1.05	0.958	0.079	12.093	***	0.795			

CHL, perceived charismatic leadership; PV, professional values; PB, professional belonging; PE, professional efficacy; WE, work engagement. B = unstandardized loading; β = standardized loading; α = Cronbach’s alpha; CR = composite reliability; AVE = average variance extracted.

***p < 0.001.

Average variance extracted ranged from 0.584 to 0.744. Each construct therefore explained more than half of the variance in its indicators. Taken together, the loading, reliability, and AVE results supported the convergent validity of the localized measures.

### 4.3 Correlations and discriminant validity

Table 6 presents latent-construct correlations, square roots of AVE, and the supplied HTMT ratios. Perceived charismatic leadership was positively correlated with professional values (r = 0.521), professional belonging (r = 0.530), professional efficacy (r = 0.412), and work engagement (r = 0.366; all *p* < 0.01). Work engagement was correlated with professional efficacy (r = 0.720), professional values (r = 0.591), and professional belonging (r = 0.567; all *p* < 0.01). These zero-order associations are descriptive and should not be confused with the unique structural coefficients reported below.

**TABLE 6:** Construct correlations and discriminant validity.

Construct	CHL	PV	PB	PE	WE
CHL	**0.764**	0.212	0.09	0.145	0.056
PV	0.521**	**0.804**	0.041	0.807	0.675
PB	0.530**	0.697**	**0.863**	0.105	0.032
PE	0.412**	0.694**	0.691**	**0.810**	0.744
WE	0.366**	0.591**	0.567**	0.720**	**0.802**

CHL, perceived charismatic leadership; PV, professional values; PB, professional belonging; PE, professional efficacy; WE, work engagement. Values below the diagonal are Pearson correlations. Values on the diagonal in bold are the square roots of the Average Variance Extracted (AVE). Values above the diagonal are the Heterotrait-Monotrait (HTMT) ratios.

**p < 0.01.

The square root of each construct’s AVE exceeded its correlations with the other constructs. The reported HTMT ratios in the upper triangle were also below the conservative 0.85 reference value (Henseler et al., 2015). The five-factor results and collapsed-model comparisons therefore support treating the constructs separately in the parallel model. The relatively high efficacy-values HTMT ratio (0.807) nevertheless indicates substantial relatedness and warrants caution against presenting the dimensions as psychologically independent.

### 4.4 Structural model fit

The structural model produced mixed but broadly adequate fit: Chi-square/df = 2.558, GFI = 0.902, CFI = 0.924, TLI = 0.909, RMSEA = 0.079, 90% CI [0.069, 0.090], and SRMR = 0.0412 (see Table 7). CFI, TLI, GFI, and SRMR met commonly used adequacy criteria, whereas the RMSEA interval did not support close fit and its upper bound reached 0.090.

**TABLE 7:** Structural model fit.

χ ^2^	df	χ ^2^/df	GFI	CFI	TLI	RMSEA [90% CI]	SRMR
327.447	128	2.558	0.902	0.924	0.909	0.079 [0.069, 0.090]	0.0412

Because fit indices are sensitive to model complexity and sample characteristics, they were interpreted jointly. The pattern was considered sufficient for cautious examination of the prespecified coefficients (see Table 7), but it does not justify describing the structural fit as uniformly strong.

### 4.5 Direct associations and hypothesis testing

As shown in Table 8, perceived charismatic leadership was not uniquely associated with work engagement after the three professional resources were included (beta = −0.075, *p* = 0.178); H1 was therefore not supported. It was positively associated with professional efficacy (beta = 0.172, *p* = 0.018) and professional values (beta = 0.233, *p* = 0.002), supporting H2a and H2c, but its coefficient for professional belonging was not significant (beta = 0.095, *p* = 0.186), so H2b was not supported.

**TABLE 8:** Structural path coefficients and hypothesis-testing results.

Hypothesized relationship	*B*	*SE*	*z*	*p*	β	Decision
H1: CHL→WE	−0.096	0.072	−1.347	0.178	−0.075	Not supported
H2a: CHL→PE	0.254	0.107	2.369	0.018	0.172	Supported
H2b: CHL→PB	0.111	0.084	1.323	0.186	0.095	Not supported
H2c: CHL→PV	0.303	0.095	3.174	0.002	0.233	Supported
H3a: PE→WE	0.687	0.068	10.155	<0.001	0.790	Supported
H3b: PB→WE	−0.045	0.056	−0.809	0.419	−0.041	Not supported
H3c: PV→WE	0.205	0.055	3.757	<0.001	0.208	Supported

CHL, perceived charismatic leadership; PE, professional efficacy; PB, professional belonging; PV, professional values; WE, work engagement. Squared multiple correlations were PE = 0.030, PB = 0.009, PV = 0.054, and WE = 0.641.

Professional efficacy had a positive unique association with work engagement (beta = 0.790, *p* < 0.001), supporting H3a. The coefficient for professional belonging was not significant (beta = −0.041, *p* = 0.419), so H3b was not supported. Professional values had a positive unique association with work engagement (beta = 0.208, *p* < 0.001), supporting H3c.

The squared multiple correlations were 0.030 for professional efficacy, 0.009 for professional belonging, 0.054 for professional values, and 0.641 for work engagement. Thus, perceived charismatic leadership alone accounted for modest proportions of variance in the three professional resources, whereas the full set of four predictors accounted for 64.1% of the variance in the adapted work-engagement construct. These values describe explained variance within the specified covariance model and should not be interpreted causally.

### 4.6 Parallel indirect-association analysis

The specific indirect estimates are reported in Table 9. The indirect association between perceived charismatic leadership and work engagement through professional efficacy was 0.175. Its bias-corrected 95% confidence interval [0.031, 0.381] and percentile interval [0.030, 0.372] excluded zero; H4a was therefore supported.

**TABLE 9:** Specific indirect associations.

Hypothesis	Indirect pathway	Indirect estimate	Bias-corrected 95% CI	Percentile 95% CI	Decision
H4a	CHL → PE → WE	0.175	[0.031, 0.381]	[0.030, 0.372]	Supported
H4b	CHL → PB → WE	−0.005	[−0.038, 0.005]	[−0.025, 0.012]	Not supported
H4c	CHL → PV → WE	0.062	[0.013, 0.152]	[0.004, 0.142]	Supported

Indirect estimates were evaluated with 5,000 bootstrap resamples. An estimate was considered statistically distinguishable from zero when its 95% confidence interval excluded zero. The estimates are cross-sectional indirect associations, not evidence of temporal or causal mediation.

The corresponding estimate through professional belonging was −0.005, and both confidence intervals included zero; H4b was not supported. The estimate through professional values was 0.062, and its bias-corrected interval [0.013, 0.152] and percentile interval [0.004, 0.142] excluded zero; H4c was supported.

Professional efficacy and professional values therefore showed statistically reliable specific indirect associations, whereas professional belonging did not. The efficacy estimate was descriptively larger than the values estimate, but no bootstrap contrast between the two was performed. The results cannot establish that efficacy is a stronger causal mechanism or that the proposed temporal sequence occurred.

## 5 Discussion

### 5.1 Principal findings

This study examined whether the covariance among perceived charismatic leadership, three professional resources, and work engagement was consistent with a parallel indirect-association model. Four findings define the pattern. First, perceived charismatic leadership did not have a significant unique association with work engagement after the proposed mediators were included. Second, it was positively associated with professional efficacy and professional values but not with professional belonging. Third, efficacy and values had positive unique associations with engagement, whereas belonging did not. Fourth, the specific indirect estimates through efficacy and values excluded zero, while the estimate through belonging did not.

The two supported indirect estimates differed descriptively: the unstandardized estimate through professional efficacy was 0.175 and that through professional values was 0.062. Because the study did not test their difference directly, the data do not establish that one route is statistically stronger than the other. The efficacy-values HTMT ratio of 0.807 further indicates that capability- and meaning-related perceptions are distinct but closely related in this sample. The defensible conclusion is therefore that each retained a unique association in the specified model, not that charismatic leadership activated a ranked causal hierarchy.

The findings are consistent with a differentiated resource-conversion account: perceived charismatic leadership covaried with engagement through capability- and meaning-related professional perceptions, but not through the unique component of school and professional-community belonging. This pattern challenges the assumption that all favorable professional perceptions make equivalent statistical contributions. It remains one of several possible temporal explanations of the observed covariance.

### 5.2 Interpreting the nonsignificant direct association

The nonsignificant CHL-to-WE coefficient indicates that perceived inspiration, competence, and enthusiasm did not explain unique variance in work engagement once efficacy, belonging, and values were included. Academic work is autonomous and specialized and is also shaped by workload, resources, evaluation systems, and institutional procedures. A teacher may evaluate a leader favorably without reporting correspondingly high day-to-day engagement.

This pattern is consistent with studies in which leadership-engagement associations were statistically linked with more proximal resources. Job autonomy accounted for the association between transformational leadership and work engagement in a university sample (Gözükara and Simsek, 2015), job demands were an indirect statistical mechanism in higher education (Atiku and Van Wyk, 2024), and psychological empowerment occupied an intervening position among Malaysian academics (Abdulrab et al., 2025). These comparisons support the plausibility of a resource-based account but do not validate the temporal ordering in the present data.

The result does not mean that perceived charismatic leadership was unrelated to the model. It had significant coefficients for two proposed mediators, which participated in nonzero specific indirect associations. The result is therefore better described as an absence of a unique direct association alongside two supported cross-sectional indirect associations.

This distinction also illustrates why direct and indirect estimates answer different questions. Estimating the professional resources simultaneously shows that a nonsignificant direct coefficient can coexist with theoretically relevant covariance carried through other variables. Because all variables were measured concurrently, reverse or reciprocal ordering remains possible: engaged teachers may evaluate leaders more favorably, and engagement may contribute to efficacy or value affirmation.

### 5.3 Professional efficacy as a capability-related indirect association

Professional efficacy had the largest unique coefficient for work engagement in the specified model (beta = 0.790), and its specific indirect estimate was 0.175. This pattern is theoretically compatible with the idea that confidence makes demanding work feel more manageable and supports persistence. It should not be interpreted as proof that perceived leadership caused efficacy or that efficacy subsequently caused engagement.

The finding converges with a growing body of educational leadership research. Self-efficacy mediated paternalistic leadership and work engagement among medical university teachers (Xie and Wang, 2025). Authentic leadership influenced teacher engagement through teacher efficacy, both separately and in combination with school climate (Shao et al., 2025). Transformational leadership and calling were connected with teacher work engagement through self-efficacy (Zhang et al., 2026), and teaching efficacy transmitted inclusive leadership to thriving among young university teachers (Gao et al., 2026).

The present analysis adds two statistical refinements. First, an efficacy-related professional perception retained a unique association when belonging and values were estimated at the same time. Second, the corresponding indirect estimate excluded zero even though the direct leadership-engagement coefficient did not. These results identify professional efficacy as a plausible capability-related route for future longitudinal testing rather than as a demonstrated causal conversion mechanism.

If longitudinal or experimental research confirms this ordering, the practical implication would be to pair inspirational communication with capability-building experiences such as useful feedback, access to resources, peer learning, manageable reform steps, and opportunities for successful performance. The present data support these practices as theory-informed possibilities, not as tested interventions.

### 5.4 Professional values as a meaning-related indirect association

Professional values also participated in a significant specific indirect association. Perceived charismatic leadership was positively associated with beliefs that teaching contributes to students, society, personal life value, and occupational respect, and these beliefs were uniquely associated with work engagement. The estimate remained nonzero after efficacy and belonging were included.

This interpretation is consistent with longitudinal research showing that value congruence can link leadership perceptions with later work engagement (Mastrorilli et al., 2026) and with mission-valence research identifying purpose and motivation as intervening conditions (Xie et al., 2026). Those designs strengthen the theoretical plausibility of meaning-related processes, but they do not convert the current cross-sectional estimate into causal evidence.

The finding is particularly relevant to higher education, where many tasks involve delayed outcomes and complex performance demands. Teachers may continue investing in teaching, advising, and institutional work when they see these activities as contributing to student development and social progress. Professional values can therefore provide motivational direction even when immediate rewards or recognition are limited. Research linking occupational values with professional quality of life also suggests that values shape how service professionals interpret demanding work (Ni et al., 2025).

The values result broadens the proposed explanation beyond capability. Efficacy addresses whether teachers believe they can perform effectively; professional values address whether the work is worth sustained effort. Both retained unique associations in the model. Subject to stronger designs, leadership development may therefore benefit from combining competence support with credible communication about educational purpose rather than relying on abstract slogans.

### 5.5 Why belonging was not a unique indirect association

Professional belonging did not show a supported specific indirect association because neither the CHL-to-PB coefficient nor the PB-to-WE coefficient was significant in the parallel model. This is not evidence that belonging is generally unimportant. At the bivariate level, belonging correlated with both perceived charismatic leadership and work engagement; the structural result indicates only that it did not explain unique variance after efficacy and values were included.

Teacher-belonging research helps explain this pattern. Belonging is created through collegial relationships, collaboration, recognition, institutional stability, professional networks, leadership support, and opportunities to contribute (Allen et al., 2025). A scoping review similarly found that collegial interaction, collective values, leadership, recognition, and teacher-student relationships jointly shape teachers’ belonging (Wator et al., 2025). Charismatic behavior by one leader may therefore be too narrow to change a resource that is embedded in the wider social system.

Voice may be especially important. Bjorklund and Karnopp (2026) showed that experiences in which teachers’ opinions were counted enhanced belonging. Inspirational communication without participatory structures may not create the same effect. In universities, teachers may also identify more strongly with disciplines, research communities, or external professional networks than with the employing institution. This possibility could weaken the link between school-level belonging and daily work engagement.

A statistical explanation is also plausible. Professional belonging correlated strongly with efficacy and values. Although the measurement comparisons support separate factors, the parallel model estimates only the unique component of each mediator. Shared positive professional orientation may have been represented more directly by efficacy and values, leaving little independent covariance for belonging. Longitudinal research should compare alternative models in which belonging precedes efficacy or value internalization, follows engagement, or participates in reciprocal relationships.

### 5.6 Theoretical implications

First, the study develops a resource-conversion interpretation of perceived charismatic leadership. The observed covariance was differentiated rather than uniform: the leadership measure was indirectly associated with engagement through resources having capability- and meaning-related content. This interpretation complements JD-R research by specifying professional resources that warrant temporal testing (Bakker and Demerouti, 2007; Rahmadani et al., 2020).

Second, the study disaggregates teacher professional identity. Efficacy, belonging, and values arose from the same broad framework, but their unique coefficients differed. Modeling them separately showed that capability- and meaning-related perceptions retained associations with engagement, whereas the unique belonging coefficient did not. A single total professional-identity score could have concealed this differentiation.

Third, the model integrates social-cognitive and value-based explanations while acknowledging Kahn’s psychological-conditions perspective. Efficacy concerns expected capability, values concern the worth of the work, and belonging has conceptual relevance to relational safety. The results are consistent with capability and meaning as plausible indirect routes, but the study did not directly measure psychological availability, meaningfulness, or safety (Kahn, 1990; May et al., 2004).

Fourth, the nonsignificant belonging route identifies a potential boundary condition for future study. Relational resources may depend on distributed social and organizational support before they are uniquely associated with engagement. Future leadership research should distinguish perceptions that may be closely tied to a focal leader from those that depend on collegial and institutional systems.

### 5.7 Practical implications

Higher education leaders should not assume that personal charisma or inspirational communication will automatically sustain teacher engagement. The present associations suggest that leadership initiatives should be evaluated by whether they are accompanied by credible capability support and professional purpose. Because no intervention was tested, the recommendations below should be treated as theory-informed applications requiring evaluation.

To support professional efficacy, department heads and academic leaders can provide timely instructional feedback, mentoring, peer demonstration, professional learning, access to teaching resources, and staged implementation of reform tasks. Recognition should focus not only on outcomes but also on evidence of growing competence. These practices are intended to provide mastery-relevant experiences and make leadership encouragement more credible.

To support professional values, leaders should connect institutional priorities with student growth, knowledge creation, social contribution, and teachers’ long-term professional development. Communication should show how administrative or reform tasks contribute to educational purposes. Evaluation and reward systems should also make socially valuable teaching and mentoring visible, rather than communicating purpose while rewarding only narrow indicators.

Belonging should be cultivated through a broader organizational strategy. Participatory decision making, psychological safety, collegial collaboration, fair workload allocation, recognition, and cross-disciplinary communities may be more relevant than charisma alone. The nonsignificant specific indirect association therefore directs future intervention research away from leader image and toward relational and institutional design.

### 5.8 Limitations and future research

Several limitations define the scope of the conclusions and the priorities for future research.

First, the cross-sectional design precludes causal inference and does not establish the temporal ordering required for mediation. The proposed leadership-to-resource-to-engagement sequence is theoretically specified, whereas the data show only a covariance pattern consistent with that sequence. Reverse and reciprocal explanations remain plausible: engaged teachers may evaluate leaders more positively, engagement may strengthen professional confidence and value affirmation, and professional resources may shape leadership perceptions. Multi-wave, diary, cross-lagged, or experimental studies are required to compare these orderings.

Second, convenience recruitment from five institutions in Shandong Province limits generalizability. Female teachers and teachers at lecturer rank or below constituted large proportions of the sample. The sample included two public undergraduate institutions, two private undergraduate institutions, and one higher vocational institution, with institution-specific sample sizes ranging from 45 to 56. Because respondents were nested within only five institutions, shared organizational climates may have reduced response independence, and institutional-level variance was not modeled. Future studies should recruit a larger number of institutions through stratified sampling, retain department-level cluster identifiers, and use multilevel or cluster-robust analyses when appropriate.

Third, the adapted measures impose construct-coverage limitations. The professional-efficacy component includes perceived suitability and responsible task enactment as well as confidence, so it is broader than task-specific teacher self-efficacy and may overlap with person-job fit or conscientious professional behavior. The four work-engagement items primarily represent sustained involvement and absorption and underrepresent dedication and the broader vigor domain; they are not equivalent to the UWES-9. The charismatic-leadership items capture follower perceptions of a culturally grounded subdimension of transformational leadership and do not establish discriminant validity against servant, authentic, spiritual, situational, or other leadership measures. Replication should use comprehensive, internationally validated measures and explicitly compare adjacent leadership constructs.

Fourth, all focal constructs came from the same self-report survey. The one-factor model was poor, but the ULMC model improved fit, indicating that method-related covariance cannot be excluded. Multi-source designs using leader reports, observed behavior, administrative indicators, or repeated assessments would reduce percept-percept inflation.

## 6 Conclusion

This study evaluated a cross-sectional parallel indirect-association model linking perceived charismatic leadership with higher education teachers’ work engagement through professional efficacy, professional belonging, and professional values. Perceived charismatic leadership did not have a significant unique direct association with work engagement, but the specific indirect estimates through professional efficacy and professional values excluded zero. The corresponding estimate through professional belonging did not.

The results support a differentiated associational account rather than a causal conclusion. Capability- and meaning-related professional perceptions retained unique relationships with engagement in the specified model, whereas the unique belonging route did not. For higher education institutions, the findings suggest hypotheses for practice: inspirational leadership may be more useful when paired with concrete capability support and credible professional-purpose practices. Longitudinal and multi-source evidence is needed before these associations are treated as mechanisms of change.

## Data Availability

The original contributions presented in the study are included in the article/supplementary material, further inquiries can be directed to the corresponding author.
